# Arthroscopy Treatment of Scaphoid Pseudarthrosis: Description of the Technique and Case Series

**DOI:** 10.1055/s-0043-1770969

**Published:** 2024-01-29

**Authors:** Ricardo Kaempf, João Brunelli, Leohnard Bayer, Márcio Aita, Santhiago Pereira Schneider, Pedro J. Delgado

**Affiliations:** 1 Santa Casa de Misericórdia de Porto Alegre, Porto Alegre, RS, Brasil; 2 Faculdade de Medicina do ABC, Santo André, SP, Brasil; 3 Hospital Universitário Madrid Montepríncipe, Universidad CEU San Pablo, Boadilla del Monte, Madri, Espanha

**Keywords:** arthroscopy, bone screws, graft, pseudarthrosis, scaphoid bone, wrist

## Abstract

**Objective** To describe the procedure and evaluate the results of a series of patients with stable and unstable pseudarthrosis of the scaphoids treated with the use of arthroscopy associated with cancellous bone graft and compression screw.

**Methods** Twenty-three patients were treated with this technique. The minimum postoperative follow-up was 12 months, and pre- and postoperative functional, clinical, and imaging analyses were performed.

**Results** Themean time from fracture to surgery was 26 months (12–60). Preoperative and postoperative clinical and radiological parameters were analyzed. The mean follow-up was 24.2 (12–60) months. Consolidation occurred in 22 patients (95.6%) in an average of 7.5 (4–12) weeks. Mean flexion range ofmotion improved from73.6° (60–80°) to 79.5° (60–90°); the range from 68.6° (50–80°) to 71.9° (45–85°); ulnar deviation from 20.6° (15–30°) to 26.9° (20–35°); and the radial deviation from 17.3° (15–25°) to 20.4° (10–25°). Pain (Visual Analog Scale [VAS] 0–10) improved from 7.3 (4–9) to 0.7 (0–6) and DASH functional scale improved from 49 (32–75) to 6 (2–12). The scapholunate angle improved from 69.1° (55–85°) to 48.4° (40–55°) and the radiolunar angle improved from 30° (10–40°) to 2.6° (0–8°).

**Conclusion** Treatment of stable and unstable pseudarthrosis of the scaphoid with spongy bone graft and percutaneous internal fixation, preferably with a headless compression screw, assisted by arthroscopy, showed good clinical and radiographic results in our series, with a short time for consolidation and functional recovery.

## Introduction

Consolidation failure is one of the main complications in the treatment of scaphoid fractures, and it can progress to a degenerative pattern called SNAC (scaphoid nonunion advanced collapse) wrist, which generates pain, stiffness and loss of strength. To prevent this serious problem, scaphoid pseudarthrosis must be treated surgically, and the classic procedure is based on debridement of the lesion focus, placement of a bone graft and rigid internal fixation. 1 2 3 4 5 6

The choice of the type of bone graft used in the treatment of scaphoid pseudarthrosis varies according to the vascularization and deformity of the scaphoid, which may be spongy for lesions with little deformity, cortico-spongy for unstable lesions and humpback deformity, and vascularized when the proximal pole of the scaphoid is necrotic. 2 3 6 7 These techniques make it possible to correct the deformity and restore the anatomy and alignment of the carpus, but most of them require a broad approach, producing damage to the capsule and ligaments, which may cause fibrosis, stiffness and loss of proprioception. 8

Recently, some authors have shown the advantages of using arthroscopy and minimally invasive procedures in the treatment of fractures and scaphoid pseudarthrosis. It highlights the lower morbidity, generating rapid recovery, in addition to allowing the treatment of associated injuries during the same procedure. In addition, wrist proprioception is preserved by minimizing damage to the capsule and ligaments and does not cause further damage to the already fragile vascularization of the scaphoid. 9 10 11 12 13 14 15

Arthroscopy was initially indicated for stable scaphoid pseudarthrosis, taking into account the difficulty of correcting the scaphoid flexion deformity without the use of a structured cortico-spongy graft. 14 However, Cohen et al. 3 , using only cancellous bone graft (unstructured) in scaphoid pseudarthrosis with collapse, had 100% consolidation and excellent clinical results with two years of evolution. As a result, some authors began to use arthroscopy in the treatment of all patterns of scaphoid pseudarthrosis, including those that were unstable and collapsed in flexion. 14 16 17 Recent articles even show good results in the treatment of scaphoid pseudarthrosis without the use of bone graft, betting on the biological regenerative potential of the wrist and bone stability. 18

We will describe here the technique and results of a series of 23 patients with scaphoid pseudarthrosis, including unstable lesions and carpal collapse, treated using arthroscopy, cancellous graft and percutaneous internal fixation, preferably with headless compression screw.

## Material and method

This work was submitted and accepted by the ethics committee of our hospital, and the term of Free, Prior and Informed Consent used was approved.

Twenty-three patients (18 men and 5 women) were operated on using this technique. The mean age was 33.4 (16-55) years and the mean time between fracture and surgery was 26 months (12-60). As for the type of initial fracture, 1 was A2, 7 were B1, 9 were B2 and 6 were B3, according to the Herbert and Fisher classification. 19 20 Among the included cases, 5 (21.7%) had fibrous union and 11 (47.8%) had humpback deformity. In three patients, a partial lesion of the scapholunate ligament was observed during arthroscopy, and two had a TFCC lesion, having been treated in the same procedure.

We present a retrospective study of a series of patients diagnosed with scaphoid pseudarthrosis treated with arthroscopy-assisted reconstruction, operated between January 2016 and October 2020. The inclusion criteria were absence of consolidation after fracture of the scaphoid at least six months ago of evolution after the initial trauma. Patients with severe degenerative changes in the radiocarpal and midcarpal joints (RC) (SNAC types II and III) were excluded. The evaluation of the vascularization of the proximal pole was performed using magnetic resonance imaging (MRI). However, vascular alteration of the scaphoid was not an exclusion criterion, nor was previous surgery at the site.

After anamnesis and physical examination suggestive of the lesion, all patients had the diagnosis confirmed with bilateral radiographs of the wrist, showing classic characteristics of scaphoid pseudarthrosis, with consolidation failure associated with sclerosis at the bone edges and cystic alterations. Before surgery, all patients underwent bilateral radiography of the wrists and computed tomography (CT) and MRI of the affected wrist.

We used the Herbert and Fischer classification preoperatively to define the type of scaphoid fracture. 19 20 ( Table 1 ). The degree of displacement and instability of the lesion, defined through the scapholunate (SL) and radiolunar (RL) angles, were obtained using PACS® system measurement tools (Terch Heim, Seoul, Republic of Korea). 4

**Table 1:** Classification of scaphoid fractures by Herbert and Fisher

Classification of scaphoid fractures by Herbert and Fisher				
	Type	Column 1	Sub-type	Location and/or injury
A	Acute stable fracture		A1	Tubercle
			A2	Nondisplaced crack in the waist
B	Acute unstable fracture		B1	Oblique
			B2	Displaced or mobile, waist
			B3	Proximal pole
			B4	Fracture-dislocation
			B5	Comminuted
C	Delayed union		C	
D	Established nonunion		D1	Fibrous
			D2	Sclerotic
	Taken from Herbert and Fisher (17)			

Following these criteria, 27 patients who underwent treatment of scaphoid pseudarthrosis with arthroscopy were found. Due to lack of adequate follow-up, four patients were excluded, and 23 patients were analyzed, who had a complete evaluation and a minimum postoperative follow-up of 12 months. The results analyzed the clinical aspect, consolidation time and time to return to physical and work activities, as well as possible complications resulting from the surgery. All surgeries were performed according to the technique described here.

Postoperatively, a radiological study of the wrist was carried out in projections specific to the scaphoid every two weeks until consolidation, including lateral, posteroanterior and oblique projections with 45° of pronation of the forearm. In case of doubt about the healing of the scaphoid, CT scans were performed every 30 days.

Range of motion was assessed with a standard goniometer and a functional assessment was performed with the DASH questionnaire. 21 These assessments were performed preoperatively and at the end of follow-up. Bone consolidation was clinically determined by the absence of pain on palpation of the scaphoid in the anatomical snuffbox and through examinations with the presence of a bone bridge between the two ends of the scaphoid pseudarthrosis in all radiographic projections or in at least two CT slices. 17 22

## Surgical technique

To access the site of the scaphoid pseudarthrosis, the ulnar midcarpal (MC) portal is used for vision (optical) and the radial MC portal as the working portal (probe, shaver, curettes, osteotomes). The site of the scaphoid pseudarthrosis is initially explored and opened using a periosteum reamer (3mm Freer), after which it is debrided with a soft tissue and bone shaver. Finally, the devitalized fibrous and bone tissue is removed with the aid of curved curettes and delicate osteotomes. We used the dry arthroscopic technique for most of the procedure, with the serum used only for joint cleaning and removal of resected tissue fragments.

After cleaning the site of the scaphoid pseudarthrosis, the next step is to correct the DISI deformity of the proximal row of the carpus, obtained with the fixation of the reduced lunate with a 1.5mm K-wire.

Afterwards, the spongy bone graft must be placed in the site of the scaphoid pseudarthrosis. We use an insulin syringe with its tip cut obliquely to facilitate the entry and positioning of the graft. The radial MC portal must be widened by a few millimeters to allow syringe entry. After placing the graft in the pseudarthrosis site, it must be impacted with the back of a curette or with a periosteum reamer. In scaphoid pseudarthrosis of the middle or distal third, when radius styloidectomy is performed through the RC 1/2 portal, this portal can also be used for cleaning the pseudarthrosis focus and placing the graft, providing direct access to the radial and volar portion of the scaphoid.

**Fig. 1:**
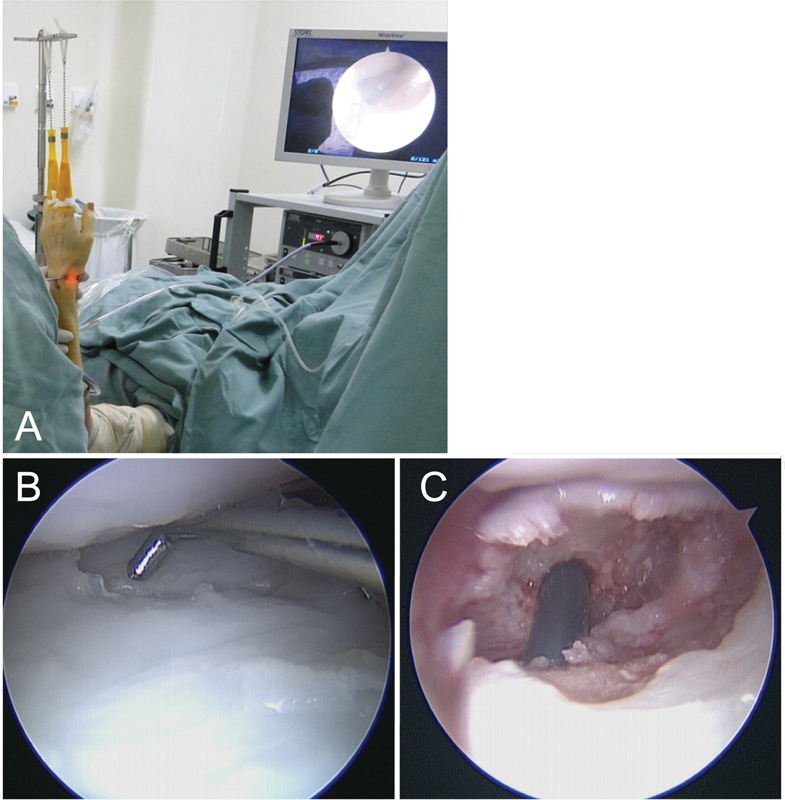
Patient with scaphoid pseudarthrosis treated using arthroscopy, cancellous bone graft and percutaneous internal fixation, using a specific traction tower and Chinese mesh placed on the second and fourth fingers (A). We used the ulnar radiocarpal portals for visualization and the radial one for instrumentation (probe) (B). After fixation, the correct position of the headless compression screw (C) can be seen.

**Fig. 2:**
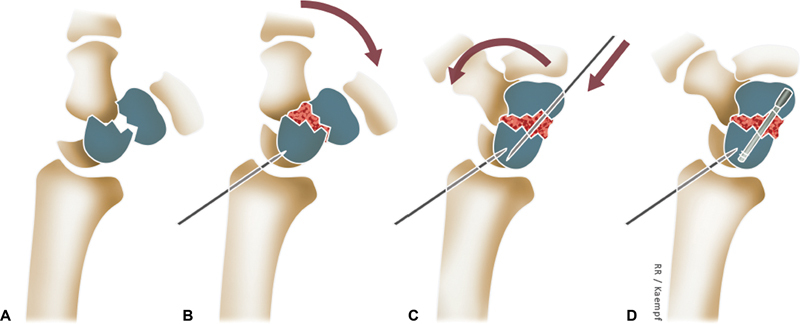
Technique for the arthroscopic treatment of scaphoid pseudarthrosis. After cleaning the edges of the pseudarthrosis, the proximal pole of the scaphoid is reduced by fixing the lunate to the radius in a neutral position (A). The bone graft is placed in the space created (B) and the wrist is reduced to the maximum extent (C) so that the distal pole of the scaphoid is reduced (1) and the scaphoid gains length. The scaphoid is then fixed with a 1.2mm Kirschener wire (2) and both scaphoid fragments are fixed with a cannulated headless screw. (D).

**Fig. 3:**
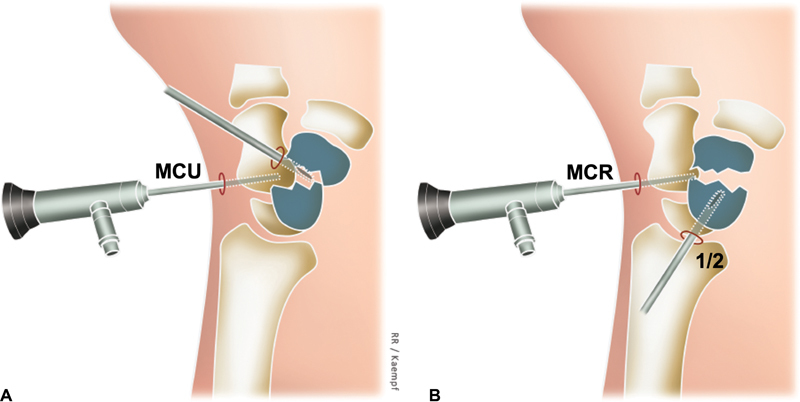
Initially, the ulnar MC portal is used for vision (optical) and the radial MC portal as the workplace (probe, shaver, curettes, osteotomes) (A). Afterwards, in pseudarthrosis of the middle third or distal scaphoid, when the radius styloidectomy is performed through the RC 1/2 portal, this portal can also be used to clean the focus of the pseudarthrosis and place the graft, providing direct access to the radial portion and scaphoid volar (B).

**Fig. 4:**
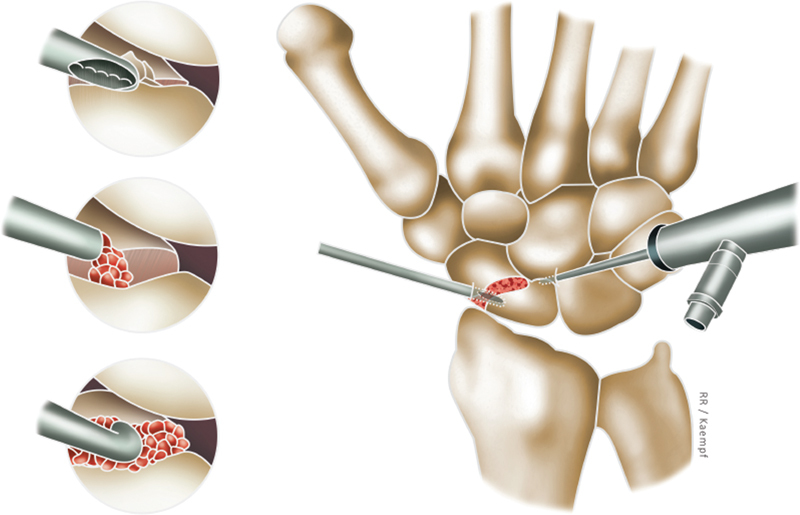
Main steps in the treatment of scaphoid pseudarthrosis using arthroscopy. Initially, the focus of the lesion is cleaned using a shaver (top detail). After that, the bone graft is placed and its impaction is performed (middle and lower detail).

**Fig. 5:**
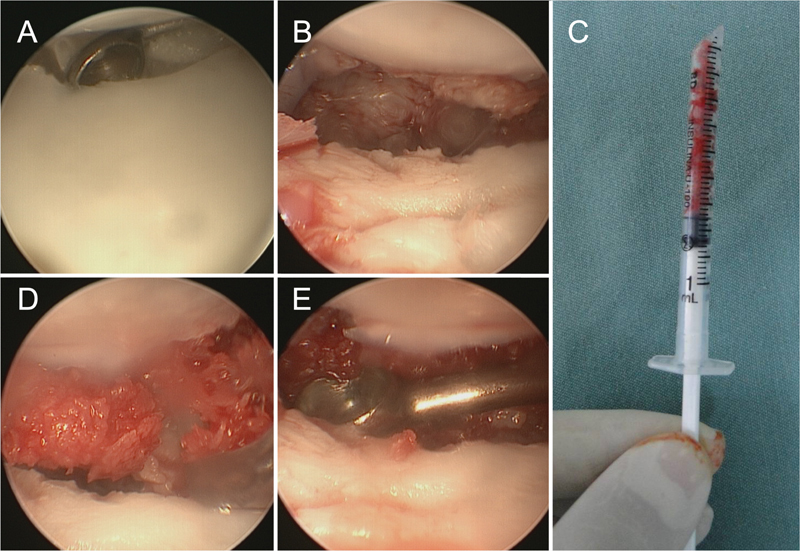
Cleaning of the scaphoid bone defect with the aid of a curette (A and B), bone graft placed in an insulin syringe (C and D) and the final appearance after impaction of the graft inside the bone defect (E).

**Fig. 6:**
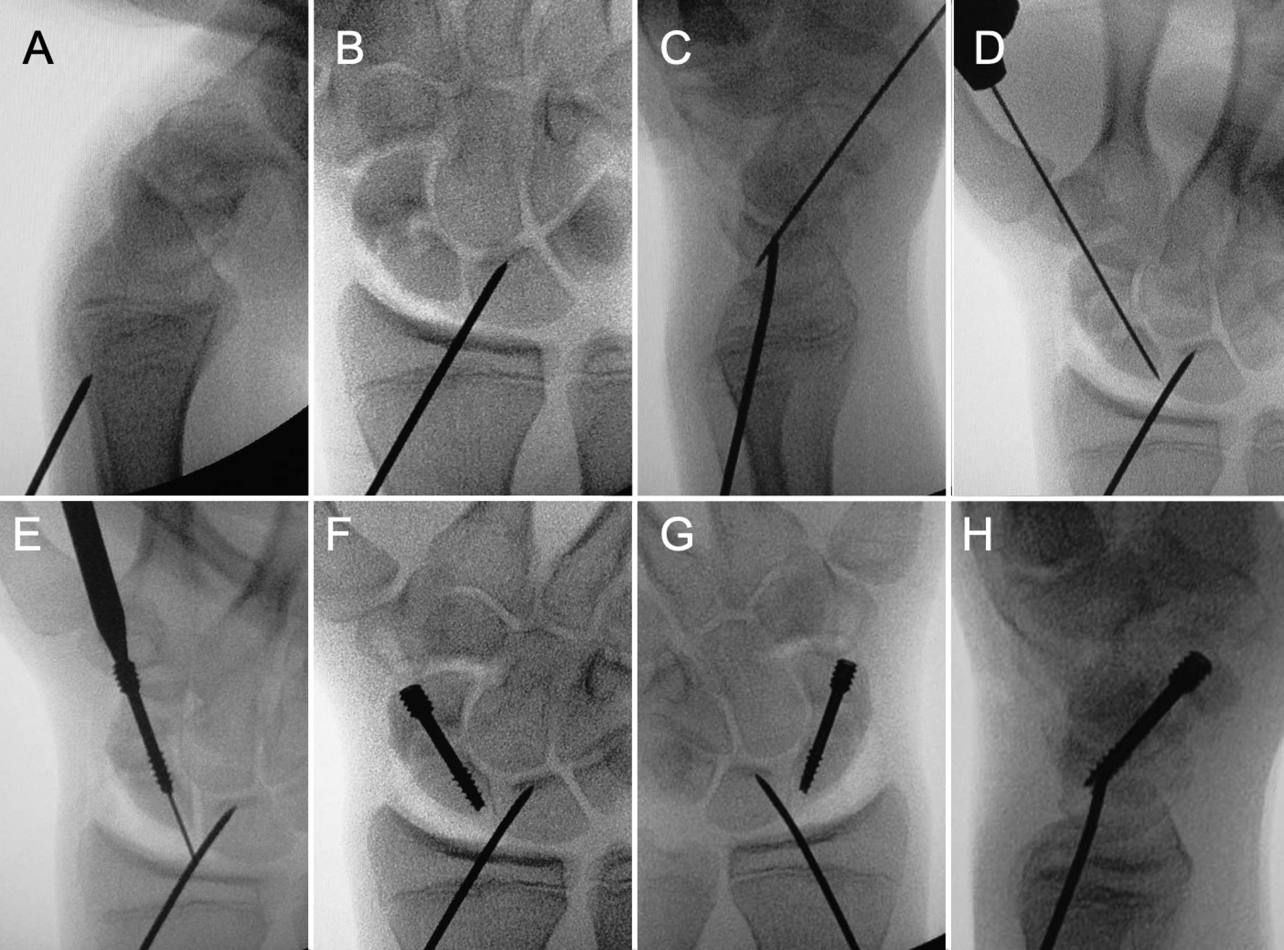
Steps of reduction of the scaphoid and restoration of carpal alignment with a Kirschner wire through the radius, fixing the lunate in a neutral position (A and B). Afterwards, placement of the guide Kirschner wire (C and D) and percutaneous fixation with the headless compression screw in a retrograde manner (E, F, G and H).

The last step of the procedure is the reduction of the distal pole and fixation of the scaphoid, preferably with the headless compression screw. Depending on the type and location of the lesion, fixation can be performed with Kirschner wires, mini-fragment screws or associated methods. For this, the handle is removed from the traction tower and placed with the palm upwards on the support table. For the reduction of the distal pole, the wrist must be positioned at maximum extension, a maneuver that also generates better alignment and gain in length of the scaphoid. We routinely use the retrograde percutaneous fixation technique for scaphoid pseudarthrosis of the middle and distal thirds. In it, a guide wire is initially used, which must enter right into the trapezium, and after checking that the wire is fixing the two fragments of the scaphoid and is central in its longitudinal axis, it must serve as a guide for the placement of the compression screw without head (cannulated Herbert or similar) ( Fig. 1 ). For scaphoid pseudarthrosis of the proximal third, we used anterograde fixation, which should be performed with the wrist in flexion and with the entry of the guide wire through the dorsum of the CR joint, so that the screw is centrally located in the longitudinal axis of the scaphoid. The correct entry point for the guide wire and subsequently for the screw can be found with the aid of arthroscopy. Under direct visualization, an abocath is placed in the proximal and ulnar part of the scaphoid, next to the insertion of the scapholunate ligament ( Figs. 2 , 3 , 4 , 5 and 6 ).

**Fig. 7:**
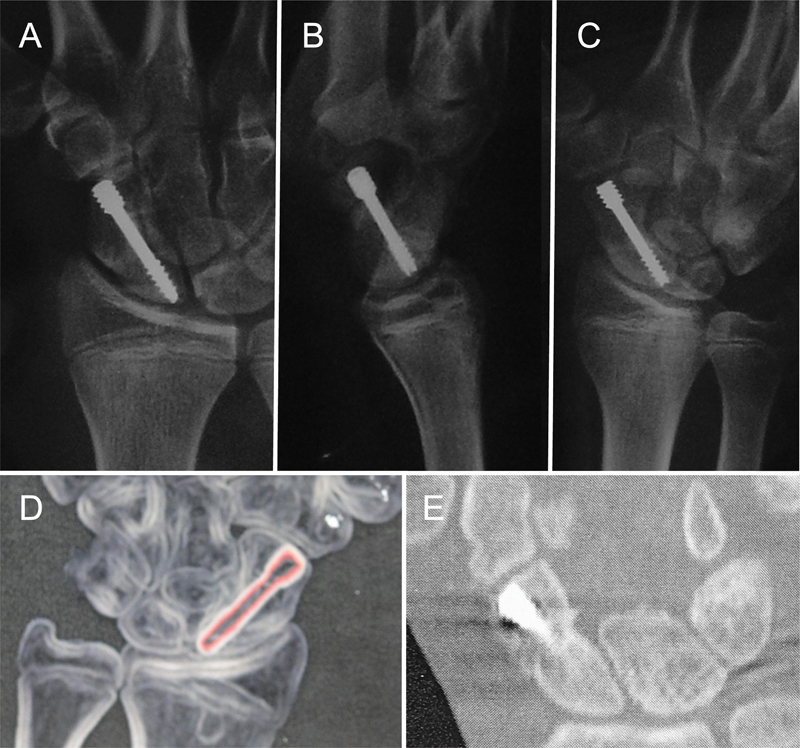
Consolidation of pseudarthrosis after five weeks of evolution. Observed by radiographs (A, B and C) and computed tomography (D and E).

We usually perform this procedure on an outpatient basis. After the surgery, the patient is immobilized with a plaster splint on the wrist (antebrachio-palmar), leaving the fingers and thumb completely free, for two weeks. We maintained the FK by fixing the radius to the lunate for four weeks, after which the splint was removed and the patient was referred to therapy. Lesions heal over an average period of seven weeks ( Fig. 7 ), and until consolidation, the patient is advised to use a removable orthosis on the wrist in activities that require strength.

## Results

Consolidation was achieved in 22 patients (95.6%) in an average of 7.5 weeks (4 to 12). As for complications, one patient did not heal and another had skin burns due to overheating of the shaver motor, which was resolved with serial dressings. None of the patients had neurotendinous injuries or there was a need to reverse the procedure using an open technique.

**Fig. 8:**
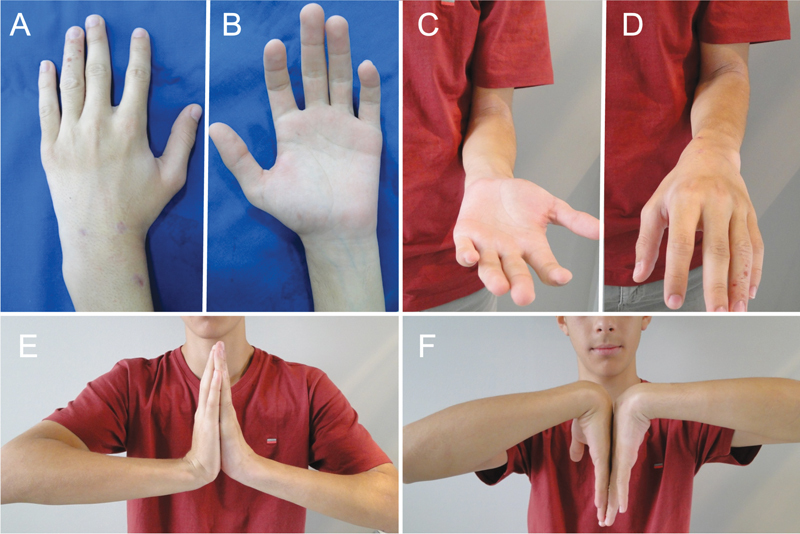
Patient eight weeks after the operation, view of the dorsal and volar face of the wrist with the incisions in the arthroscopy portals and percutaneous fixation of the scaphoid (A and B). Complete recovery of mobility, both in pronation-supination (C and D) and in extension and flexion (E and F).

The mean follow-up was 24.2 months (12-60) months. The degree of mobility, pain, functional assessment (DASH questionnaire) and radiographic measurements improved in relation to the preoperative period, being shown in Tables 2 and 3 . Mean flexion range of motion improved from 73.6° (60-80°) to 79.5° (60-90°), extension from 68.6° (50-80°) to 71.9° (45°). -85°), ulnar deviation 20.6° (15-30°) to 26.9° (20-35°) and radial deviation 17.3° (15-25°) to 20.4° (10-25°) . Pain (VAS 0-10) improved from 7.3 (4-9) to 0.7 (0-6), and the DASH functional scale improved from 49 (32-75) to 6 (2-12). The scapholunate angle improved from 69.1° (55-85°) to 48.4° (40-55°) and the radiolunar angle improved from 30° (10-40°) to 2.6° (0- 8°) ( Fig. 8 ).

**Table 2:** Preoperative findings of the casuistry of the article

Patient	Gender	Age	Side	Dominance	Pseudarthrosis time (months)	Class. by Herbert	SLA	RLA	Pain	Flex.	Ext.	RD	UD	DASH
1	M	28	R	R	12	B1	60	20	8	75	70	20	30	60
2	M	16	R	R	12	A2	65	25	4	80	75	25	25	32
3	F	55	R	R	36	B1	60	20	7	65	70	15	20	46
4	M	51	L	R	48	B2	80	40	8	65	60	15	15	50
5	M	20	R	L	18	B3	55	10	9	70	65	20	15	38
6	M	34	L	L	24	B2	80	20	8	70	60	20	20	72
7	M	29	R	R	16	B1	70	20	7	80	70	20	25	63
8	M	37	R	R	36	B2	75	30	9	75	75	15	20	57
9	F	42	R	R	20	B2	75	25	9	60	65	15	20	75
10	F	24	R	R	24	B2	85	15	8	75	70	20	15	46
11	M	25	L	R	12	B3	70	25	7	70	60	15	20	52
12	M	28	R	R	20	B3	60	10	6	80	70	20	30	36
13	M	45	L	R	36	B2	70	20	7	80	75	15	20	39
14	M	32	R	R	12	B1	75	30	8	70	75	15	20	52
15	M	17	R	R	18	B1	60	15	6	80	70	20	20	41
16	M	47	R	R	48	B2	70	20	9	75	70	15	20	64
17	M	49	L	L	42	B3	60	15	8	70	65	15	20	48
18	M	38	R	R	18	B1	65	25	7	80	70	20	25	37
19	M	37	R	R	24	B3	70	20	7	70	60	15	20	61
20	M	31	R	L	20	B3	60	10	5	80	75	15	20	42
21	F	21	R	R	60	B1	80	20	8	70	70	15	20	50
22	M	36	R	L	18	B2	75	10	8	75	75	20	15	42
23	F	28	R	R	24	B2	70	15	7	80	80	15	20	46

Abbreviations: class., classification; ext., extension; flex., flexion; RD, radial deviation; RLA, radioulnar angle; SLA, scapholunate angle; UD, ulnar deviation.

**Table 3:** Trans- and postoperative findings of the casuistry of the article

Patient	Gender	Age	Pain	SLA	RLA	Flex.	Ext.	RD	UD	Graft	Screw	Consol. (weeks)	DASH	Return to work (weeks)
1	M	28	1	50	0	80	70	20	35	IL	Her.	8	8	6
2	M	16	0	45	5	80	75	25	25	IL	Her.	6	5	4
3	F	55	1	50	5	70	70	15	20	IL	Her.	8	6	8
4	M	51	2	50	0	75	70	15	15	IL	Her.	10	7	12
5	M	20	1	45	5	80	65	20	25	IL	Her.	8	3	10
6	M	34	0	50	0	70	60	25	30	IL	Her.	10	12	8
7	M	29	0	45	5	85	75	20	30	IL	Her.	7	11	12
8	M	37	0	50	0	80	80	25	30	Rad.	ACCU	10	2	12
9	F	42	2	50	3	70	65	20	25	IL	Her.	8	9	10
10	F	24	1	55	5	80	75	25	20	IL	Her.	12	6	4
11	M	25	0	45	0	85	80	20	35	IL	Her.	10	4	4
12	M	28	0	45	0	90	80	25	30	Rad.	NORMAL	12	2	6
13	M	45	0	50	5	90	85	20	30	IL	Her.	8	8	8
14	M	32	1	55	0	75	75	20	35	IL	Her.	10	6	4
15	M	17	0	40	0	90	80	25	30	IL	Her.	8	2	10
16	M	47	2	55	5	75	65	10	20	Rad.	Her. + K-wire	16	7	12
17	M	49	1	50	0	65	70	15	25	IL	Her.	12	10	16
18	M	38	0	45	5	80	80	20	30	IL	Her.	8	4	12
19	M	37	1	50	5	75	60	20	25	IL	Her.	10	11	4
20	M	31	0	45	0	90	75	20	30	Rad.	MINI	12	8	10
21	F	21	2	50	5	80	70	20	25	IL	Her.	10	6	12
22	M	36	2	50	0	80	70	25	25	Rad.	Her.	12	3	8
23	F	28	1	45	0	90	85	20	30	Rad.	ACCU	8	2	6

Abbreviations: ACCU, Accutrak; consol., time until consolidation; ext., extension; flex., flexion; Her., Herbert; IL, iliac crest; mini, mini-fragment screw; rad., radio; RD, radial deviation; RLA, radioulnar angle; SLA, scapholunate angle; UD, ulnar deviation.

## Discussions

Other studies have already shown good results using minimally invasive methods in the treatment of fractures and scaphoid pseudarthrosis. They are associated with lower morbidity, fast recovery and allow the treatment of associated injuries during the same procedure. 6 9 14 17 23

When untreated, scaphoid pseudarthrosis generates instability that progresses to an early degenerative process, with carpal collapse, a pattern known as SNAC wrist. 1 5 Thus, the objective of the surgical treatment of scaphoid pseudarthrosis is to achieve bone consolidation associated with restoration of bone anatomy and carpal alignment. For this, techniques with different concepts have been described, some that prioritize mechanical bone stability, including the use of plates and screws, and others that aim to maintain and stimulate vascular biology, stimulating early healing. 7 14 16 18 24

Arthroscopy was initially indicated for stable and fibrous scaphoid pseudarthrosis, however, with the evolution of the technique and the confirmation that there is no need for a structural graft to correct the deformity in scaphoid flexion, its use was expanded to unstable scaphoid pseudarthrosis and with flexion collapse. In chronic and unstable scaphoid pseudarthrosis, techniques using cortico-spongy bone grafts (vascularized or not) offer the greatest guarantee of obtaining bone consolidation and restoring the alignment and anatomy of the carpus. However, as they are open techniques, they generate aggression to local tissues and neuro-vascular structures, and may also damage the extrinsic stabilizing ligaments of the carpus, in addition to the risk of stiffness, infection, graft protrusion and delay in achieving complete consolidation, a time that varies in the literature between 12 to 19 weeks. 2 3 6

Trying to reduce the trauma to local tissues and improve the consolidation potential, some articles describe good results with the use of cancellous bone graft in unstable scaphoid pseudarthrosis. Park et al. 22 treated 61 patients with scaphoid pseudarthrosis, 52% stable and 48% unstable, using cancellous bone graft and fixation with Kirschner wires. They achieved consolidation in 88.2% of stable and 83.9% of unstable lesions, with no statistically significant differences between groups.

There is no rule as to the type of bone graft to be used. We prefer the use of an iliac graft in patients with severe deformity and bone resorption. In cases of stable scaphoid pseudarthrosis with little deformity, we used a spongy bone graft taken from the distal metaphysis of the radius. The short time to achieve consolidation, mean of 7.5 weeks, is lower than those presented in series using the open technique, both in vascularized and non-vascularized grafts, allowing for a short time of immobilization and early return to activities.

Some series show results similar to ours with the use of arthroscopy and percutaneous screw fixation, without using bone graft. 9 13 14 23 25 Slade et al. 13 published a series of 15 patients with stable scaphoid pseudarthrosis achieving consolidation in all, in a mean time of 14 weeks. Chu e Shih, 26 on the other hand, treated 15 stable scaphoid pseudarthrosis patients with arthroscopy associated with combined percutaneous screw fixation and use of demineralized bone matrix. They showed 93% consolidation over 15 weeks on average. In the series by Wong and Ho with 68 patients with scaphoid pseudarthrosis treated with the arthroscopic technique and screws, there was 91.2% of union, in an average period of 12 weeks, without presenting any complications resulting from the method. 10 Kim et al. 17 evaluated 36 patients with arthroscopy and cancellous bone graft for unstable scaphoid pseudarthrosis achieving healing in 86% of patients at an average of 11 weeks.

With the arthroscopic technique using cancellous bone graft, it is more difficult to restore the anatomy of the scaphoid and the normal alignment of the carpus. However, other techniques also show similar difficulty. Jiranek et al. 27 in a series of patients treated for scaphoid pseudarthrosis with trapezoidal corticocancellous bone graft, obtained a deformity of 45° or more at the intrascaphoid angle in more than half of the cases and did not find any relationship between the alignment and the final clinical result. Also from Kim et al. 17 series, radiological parameters of the scaphoid and carpal alignment did not correlate with the patient's final clinical function. Like these authors, we think that the positive effects of the arthroscopic technique and percutaneous fixation outweigh a possible difficulty in completely correcting the deformity, given that this does not change the final function of the wrist. 28

Wrist arthroscopy is a valid alternative in the treatment of carpal scaphoid injuries. It presents good clinical and consolidation results, even in lesions with deformity and carpal instability, with a shorter recovery period. It is likely limited to restoring normal carpal alignment, but it is beneficial and improves recovery time for these injuries.

## Conclusion

The presented results demonstrate low morbidity and fast recovery with this technique. However, to confirm these results, we need larger series, with longer follow-up and with patients randomized into groups, comparing different techniques.
