# Supplementary material for: Arthroscopy Treatment of Scaphoid Pseudarthrosis: Description of the Technique and Case Series
Source: Rev Bras Ortop (Sao Paulo). 2024 Jan 29;59(5):e719–29. doi: 10.1055/s-0043-1770969 (PMC11624938; doi:10.1055/s-0043-1770969)
Supplement: Related texts

# Research article: Tratamento da pseudoartrose do escafoide com uso de artroscopia: Descrição da técnica e série de casos

**Keywords:** artroscopia, enxertos, osso escafoide, parafusos ósseos, pseudartrose, punho

## Abstract

**Objetivo** Descrever o procedimento e avaliar os resultados de uma série de pacientes com pseudartroses estáveis e instáveis dos escafoides tratados com o uso de artroscopia associado a enxerto ósseo esponjoso e parafuso de compressão.

**Método** Foram tratados 23 pacientes com esta técnica. O acompanhamento mínimo no pós-operatório foi de 12 meses, e foram realizadas análises funcionais, clínicas e de imagem pré e pós-operatórias.

**Resultados** O tempo médio desde a fratura até a cirurgia foi de 26 meses (12-60). Parâmetros clínicos e radiológicos pré-operatório e pós-operatório foram analisados. O seguimento médio foi de 24,2 (12-60) meses. A consolidação ocorreu em 22 pacientes (95,6%) em uma média de 7,5 (4-12) semanas. A amplitude média de movimento de flexão melhorou de 73,6° (60-80°) a 79,5° (60-90°); a extensão de 68,6° (50-80°) a 71,9° (45-85°); o desvio ulnar de 20,6°(15-30°) a 26,9° (20-35°); e o desvio radial de 17,3° (15-25°) em 20,4° (10-25°). A dor (Escala Visual Analógica [EVA] 0-10) melhorou de 7,3 (4-9) para 0,7 (0-6) e a escala funcional DASH melhorou de 49 (32-75) para 6 (2-12). O ângulo escafolunar melhorou de 69,1°(55-85°) para 48,4° (40-55°) e o ângulo radiolunar melhorou de 30°(10-40°) a 2,6° (0-8°).

**Conclusão** O tratamento das pseudartrose estáveis e instáveis do escafoide com enxerto ósseo esponjoso e fixação interna percutânea, preferencialmente com parafuso de compressão sem cabeça, assistidos por artroscopia, mostrou bons resultados clínicos e radiográficos na nossa casuística, com curto tempo de consolidação e recuperação funcional.

## Introdução

A falha de consolidação é uma das principais complicações no tratamento da fratura do escafoide, e pode evoluir para um padrão degenerativo chamado de punho SNAC ( *scaphoid nonunion advanced collapse* ), que gera dor, rigidez e perda de força. Para prevenir esse grave problema, as pseudartroses do escafoide (PAE) devem ser tratadas de forma cirúrgica, sendo que o procedimento clássico se baseia no desbridamento do foco da lesão, colocação de enxerto ósseo e fixação interna rígida. 1 2 3 4 5 6

A escolha do tipo de enxerto ósseo usado no tratamento das PAE varia conforme a vascularização e a deformidade do escafoide, podendos ser esponjoso para lesões com pouca deformidade, córtico-esponjoso para lesões instáveis e com deformidade em corcova ( *humpback deformity* ) e vascularizado quando o pólo proximal do escafoide está necrótico. 2 3 6 7 Essas técnicas permitem corrigir a deformidade e restaurar a anatomia e o alinhamento do carpo, porém a maioria necessita de abordagem ampla, produzindo danos à cápsula e ligamentos, podendo causar fibrose, rigidez e perda da propriocepção. 8

Recentemente alguns autores mostraram as vantagens do uso da artroscopia e de procedimentos minimante invasivos no tratamento de fraturas e PAE. Destaca-se a menor morbidade, gerando rápida recuperação, além de permitir o tratamento de lesões associadas durante o mesmo procedimento. Além disso, preserva-se a propriocepção do punho ao minimizar o dano à cápsula e ligamentos e não causa maior prejuízo à já frágil vascularização do escafoide. 9 10 11 12 13 14 15

A artroscopia foi inicialmente indicada para as PAE estáveis, levando em conta a dificuldade da correção da deformidade em flexão do escafoide sem o uso de enxerto estruturado córtico-esponjoso. 14 No entanto, Cohen et al. 3 , utilizando apenas enxerto esponjoso (não estruturado) em PAE com colapso, tiveram 100% de consolidação e ótimos resultados clínicos com dois anos de evolução. Com isso, alguns autores começaram a utilizar a artroscopia no tratamento de todos os padrões de PAE, inclusive nas instáveis e com colapso em flexão. 14 16 17 Artigos recentes mostram inclusive bons resultados no tratamento da PAE sem o uso de enxerto ósseo, apostando no potencial biológico regenerativo do punho e na estabilidade óssea. 18

Descreveremos aqui a técnica e os resultados de uma série de 23 pacientes com PAE tratados com o uso da artroscopia, enxerto esponjoso e fixação interna percutânea, preferencialmente com parafuso de compressão sem cabeça, incluindo lesões instáveis e com colapso carpal.

## Material e método

Este trabalho foi submetido e aceito pelo comitê de ética do nosso hospital, sendo aprovados os TCLE aplicados.

Vinte e três pacientes (18 homens e cinco mulheres) foram operados com esta técnica. A média de idade foi de 33,4 (16-55) anos e o tempo médio entre a fratura e a cirurgia foi de 26 meses (12-60). Quanto ao tipo da fratura inicial, uma era A2, sete B1, nove B2 e seis B3, pela classificação de Herbert e Fisher. 19 20 Entre os casos incluídos 5 (21,7%) apresentavam união fibrosa e 11 (47,8%) apresentavam *humpback deformity.* Em três pacientes foi observada durante a artroscopia uma lesão parcial do ligamento escafo-semilunar e dois apresentavam lesão da FCT, tendo sido tratadas no mesmo procedimento.

Apresentamos um estudo retrospectivo de uma série de pacientes com diagnóstico de PAE tratados com reconstrução assistida por artroscopia, operados no período entre janeiro de 2016 e outubro de 2020. Os critérios de inclusão foram ausência de consolidação após fratura do escafoide com pelo menos seis meses de evolução após o trauma inicial. Excluíram-se os pacientes com alterações degenerativas graves na articulação radiocarpal e mediocarpal (RC) (SNAC tipo II e III). A avaliação da vascularização do pólo proximal foi realizada através de Ressonância Magnética. Entretanto, alteração vascular do escafoide não foi critério de exclusão, tampouco cirurgia prévia no local.

Após anamnese e exame físico sugestivas da lesão, todos pacientes tiveram o diagnóstico confirmado com radiografias bilaterais do punho, apresentando características clássicas de PAE, com falha de consolidação associada à esclerose nos bordos ósseos e alterações císticas. Antes da cirurgia todos os pacientes realizaram radiografia bilateral dos punhos e tomografia computadorizada (TC) e ressonância magnética (RM) do punho afetado.

Utilizamos a classificação de Herbert e Fischer no pré-operatório para definir o tipo de fratura de escafoide. 19 20 ( Tabela 1 ). O grau de deslocamento e instabilidade da lesão, definidos através dos ângulos escafolunar (EL) e radiolunar (RL), foram obtidos através de ferramentas de medição do sistema PACS® (Terch Heim, Seul, República da Coréia). 4

**Tabela 1:** Classificação de Herbert e Fischer para fraturas do escafoide

Classificação da fratura do escafoide por Herbert e Fisher				
	Tipo	Coluna1	Subtipo	Localização e/ou lesão
A	Fratura aguda e estável		A1	Tuberosidade
			A2	Não deslocada do colo
B	Aguda, instável		B1	Oblíqua
			B2	Deslocada ou móvel, do colo
			B3	Pólo proximal
			B4	Fratura-luxação
			B5	Cominuta
C	Retardo de consolidação		C	
D	Pseudartrose estabelecida		D1	Fibrosa
			D2	Esclerótica
	Retirado de Herbert e Fisher (17)			

Seguindo esses critérios foram encontrados 27 pacientes submetidos ao tratamento de PAE com artroscopia. Por falta de acompanhamento adequado, quatro pacientes foram excluídos, sendo analisados 23 pacientes, que tiveram avaliação completa e acompanhamento pós-operatório mínimo de 12 meses. Nos resultados foram analisados o aspecto clínico, o tempo de consolidação e o tempo de retorno às atividades físicas e laborais, assim como possíveis complicações decorrentes da cirurgia. Todas as cirurgias foram realizadas conforme a técnica aqui descrita.

No pós-operatório, realizou-se estudo radiológico do punho em projeções próprias para o escafoide a cada duas semanas até a consolidação, incluindo laterais, póstero-anteriores e oblíqua com 45° de pronação do antebraço. Em caso de dúvida na consolidação do escafoide foram realizadas TC a cada 30 dias.

A amplitude de movimento foi avaliada com um goniômetro padrão e uma avaliação funcional foi realizada com o questionário DASH. 21 Essas avaliações foram realizadas no pré-operatório e ao final do seguimento. A consolidação óssea foi determinada clinicamente pela ausência de dor a palpação do escafoide na tabaqueira anatômica e através dos exames com a presença de ponte óssea entre as duas extremidades da PAE em todas as projeções radiográficas ou em pelo menos em dois cortes da TC. 17 22

## Técnica cirúrgica

Para acesso ao foco da PAE, utiliza-se o portal MC ulnar para visão (óptica) e o portal MC radial como portal de trabalho (probe, *shaver* , curetas, osteótomos). O foco da PAE é inicialmente explorado e aberto com o uso de um descolador de periósteo ( *Freer* de 3mm), sendo após desbridado com *shaver* de partes moles e ósseo. Por último, o tecido fibroso e ósseo desvitalizado é removido com o auxílio de curetas curvas e osteótomos delicados. Utilizamos na maior parte do procedimento a técnica artroscópica seca, sendo o soro utilizado apenas para a limpeza articular e retirada de fragmentos dos tecidos ressecados.

Após a limpeza do foco da PAE, o próximo passo é a correção da deformidade em DISI da fileira proximal do carpo, obtida com a fixação do semilunar reduzido com fio de K 1,5mm.

Depois, deve-se colocar o enxerto ósseo esponjoso no foco da PAE. Utilizamos uma seringa de insulina com a sua ponta cortada de forma oblíqua para facilitar a entrada e o posicionamento do enxerto. O portal MC radial deve ser alargado em poucos milímetros para permitir a entrada de seringa. Após a colocação do enxerto no foco da pseudartrose, esse deve ser impactado com o dorso de uma cureta ou com o descolador de periósteo. Nas PAE do terço médio ou distal, quando se realiza a estiloidectomia do rádio pelo portal RC 1/2, esse portal também pode ser utilizado para limpeza do foco da pseudartrose e colocação do enxerto, proporcionando um acesso direto a porção radial e volar do escafoide.

A última etapa do procedimento é a redução do pólo distal e a fixação do escafoide, preferencialmente com o parafuso de compressão sem cabeça. Dependendo do tipo e local da lesão a fixação pode ser feita com fios de Kirschner, parafusos de mini-fragmentos ou associando métodos. Para isso o punho é removido da torre de tração e colocado com a palma para cima sobre a mesa de apoio. Para a redução do pólo distal, o punho deve ser posicionado em máxima extensão, manobra que também gera melhora do alinhamento e ganho de comprimento do escafoide. Utilizamos de rotina a técnica de fixação percutânea retrógrada para as PAE dos terços médio e distal **.** Nela, utiliza-se inicialmente um fio guia que deve entrar justo ao trapézio, e após conferir que o fio está fixando os dois fragmentos do escafoide e está central em seu eixo longitudinal, ele deverá servir de guia para a colocação do parafuso de compressão sem cabeça (Herbert canulado ou similar) ( Fig. 1 ). Para as PAE do terço proximal utilizamos a fixação anterógrada, que deve ser realizada com o punho em flexão e com a entrada do fio guia pelo dorso da articulação RC, para que o parafuso se localize de forma central no eixo longitudinal do escafoide. O ponto correto de entrada do fio guia e posteriormente do parafuso pode ser encontrado com auxílio da artroscopia. Sob visualização direta, coloca-se um *abocath* na parte proximal e ulnar do escafoide, ao lado da inserção do ligamento escafo-semilunar, assim, com o *abocath* fixo no escafoide proximal, passa-se o FK guia por dentro dele ( Figs. 2 , 3 , 4 , 5 e 6 ).

**Fig. 1:** Paciente com pseudartrose de escafoide tratado com o uso da artroscopia, enxerto esponjoso e fixação interna percutânea, utilizando torre de tração específica e malha chinesa colocadas no segundo e quarto dedos (A). Utilizamos os portais rádio-cárpicos ulnar para visualização e o radial para instrumentação (probe)(B). Após a fixação se consegue visualizar a correta posição do parafuso de compressão sem cabeça (C).

**Fig. 2:** Técnica de tratamento da pseudartrose do escafoide com auxílio da artroscopia. Após a limpeza dos bordos da pseudartrose, o pólo proximal do escafoide é reduzido com a fixação do semilunar ao rádio em posição neutra (A). O enxerto ósseo é colocado no espaço criado (B) e o punho é reduzido em máxima extensão (C) para que o pólo distal do escafoide se reduza (1) e o escafoide ganhe comprimento. O escafoide é então fixo com um fio de Kirschener de 1,2mm (2) e ambos os fragmentos do escafoide são fixados com um parafuso canulado sem cabeça (D).

**Fig. 3:** Inicialmente se utiliza o portal MC ulnar para visão (óptica) e o portal MC radial como local de trabalho (probe, *shaver* , curetas, osteótomos) (A). Após, nas pseudartroses do escafoide do terço médio ou distal, quando se realiza a estiloidectomia do rádio pelo portal RC 1/2, esse portal também pode ser utilizado para limpeza do foco da pseudartrose e colocação do enxerto, proporcionando um acesso direto a porção radial e volar do escafoide (B).

**Fig. 4:** Principais passos do tratamento da pseudartrose do escafoide com uso de artroscopia. Inicialmente se faz a limpeza do foco da lesão com o uso de *shaver* (detalhe superior). Após se coloca o enxerto ósseo e realiza-se a sua impacção (detalhe médio e inferior).

**Fig. 5:** Limpeza do defeito ósseo do escafoide com auxílio de uma cureta (A e B), enxerto ósseo colocado em uma seringa de insulina (C e D) e o aspecto final após a impacção do enxerto no interior do defeito ósseo (E).

**Fig. 6:** Etapas da redução do escafoide e restauração do alinhamento do carpo com fio de Kirschner através do rádio fixando o semilunar em posição neutra (A e B). Após, colocação do fio de Kirschner guia (C e D) e fixação percutânea com o parafuso de compressão sem cabeça de forma retrógrada (E, F, G e H).

Realizamos esse procedimento habitualmente em regime ambulatorial. Após a cirurgia, o paciente é imobilizado com uma tala gessada de punho (antebráquio-palmar), deixando os dedos e o polegar completamente livres, por duas semanas. Mantemos o FK fixando o rádio ao semilunar por quatro semanas, sendo após esse período retirada a tala e o paciente encaminhado para terapia. As lesões consolidam em um período médio de sete semanas ( Fig. 7 ), sendo que até a consolidação, o paciente é orientado a usar uma órtese removível no punho em atividades que exijam força **.**

**Fig. 7:** Consolidação da pseudartrose com cinco semanas de evolução. Observado pelas radiografias (A, B e C) e tomografia computadorizada (D e E).

## Resultados

A consolidação foi obtida em 22 pacientes (95,6%) em uma média de 7,5 semanas (4 a 12). Quanto às complicações, um paciente não consolidou e outro apresentou queimadura cutânea por hiperaquecimento do motor do *shaver* , resolvendo com curativos seriados. Em nenhum paciente houve lesões neuro-tendinosas ou houve a necessidade de reverter o procedimento em uma técnica aberta.

O seguimento médio foi de 24,2 meses (12-60) meses. O grau de mobilidade, dor, avaliação funcional (questionário DASH) e medidas radiográficas melhoraram com relação ao pré-operatório, sendo mostrados nas Tabelas 2 e 3 . A amplitude média de movimento de flexão melhorou de 73,6° (60-80°) para 79,5° (60-90°), extensão de 68,6° (50-80°) a 71,9° (45-85°), desvio ulnar 20,6°(15-30°) a 26,9° (20-35°) e desvio radial 17.3° (15-25°) em 20,4° (10-25°). Dor (EVA 0-10) melhorou de 7,3 (4-9) para 0,7 (0-6), e a escala funcional DASH melhorou de 49 (32-75) para 6 (2-12). O ângulo escafolunar melhorou de 69,1°(55-85°) para 48,4° (40-55°) e o ângulo rádio-lunar melhorou de 30°(10-40°) para 2,6° (0-8°) ( Fig. 8 ).

**Tabela 2:** Achados pré-operatórios da casuística do artigo

Paciente	Gênero	Idade	Lado	Dominância	Tempo de pseudartrose (meses)	Class. de Herbert	SLA	RLA	Dor	Flex.	Ext.	RD	UD	DASH
1	M	28	D	D	12	B1	60	20	8	75	70	20	30	60
2	M	16	D	D	12	A2	65	25	4	80	75	25	25	32
3	F	55	D	D	36	B1	60	20	7	65	70	15	20	46
4	M	51	E	D	48	B2	80	40	8	65	60	15	15	50
5	M	20	D	E	18	B3	55	10	9	70	65	20	15	38
6	M	34	E	E	24	B2	80	20	8	70	60	20	20	72
7	M	29	D	D	16	B1	70	20	7	80	70	20	25	63
8	M	37	D	D	36	B2	75	30	9	75	75	15	20	57
9	F	42	D	D	20	B2	75	25	9	60	65	15	20	75
10	F	24	D	D	24	B2	85	15	8	75	70	20	15	46
11	M	25	E	D	12	B3	70	25	7	70	60	15	20	52
12	M	28	D	D	20	B3	60	10	6	80	70	20	30	36
13	M	45	E	D	36	B2	70	20	7	80	75	15	20	39
14	M	32	D	D	12	B1	75	30	8	70	75	15	20	52
15	M	17	D	D	18	B1	60	15	6	80	70	20	20	41
16	M	47	D	D	48	B2	70	20	9	75	70	15	20	64
17	M	49	E	E	42	B3	60	15	8	70	65	15	20	48
18	M	38	D	D	18	B1	65	25	7	80	70	20	25	37
19	M	37	D	D	24	B3	70	20	7	70	60	15	20	61
20	M	31	D	E	20	B3	60	10	5	80	75	15	20	42
21	F	21	D	D	60	B1	80	20	8	70	70	15	20	50
22	M	36	D	E	18	B2	75	10	8	75	75	20	15	42
23	F	28	D	D	24	B2	70	15	7	80	80	15	20	46

Abreviaturas: Class., classificação; ext., extensão; flex., flexão; PSA, pseudartrose; RD, desvio radial; RLA, ângulo radioulnar; SLA, ângulo escafolunar; UD, desvio ulnar.

**Tabela 3:** Achados trans e pós-operatórios da casuística do artigo

Paciente	Gênero	Idade	Dor	SLA	RLA	Flex.	Ext.	RD	UD	Enxerto	Parafuso	Consol. (semanas)	DASH	Retorno ao trabalho (semanas)
1	M	28	1	50	0	80	70	20	35	IL	Her.	8	8	6
2	M	16	0	45	5	80	75	25	25	IL	Her.	6	5	4
3	F	55	1	50	5	70	70	15	20	IL	Her.	8	6	8
4	M	51	2	50	0	75	70	15	15	IL	Her.	10	7	12
5	M	20	1	45	5	80	65	20	25	IL	Her.	8	3	10
6	M	34	0	50	0	70	60	25	30	IL	Her.	10	12	8
7	M	29	0	45	5	85	75	20	30	IL	Her.	7	11	12
8	M	37	0	50	0	80	80	25	30	Rad.	ACCU	10	2	12
9	F	42	2	50	3	70	65	20	25	IL	Her.	8	9	10
10	F	24	1	55	5	80	75	25	20	IL	Her.	12	6	4
11	M	25	0	45	0	85	80	20	35	IL	Her.	10	4	4
12	M	28	0	45	0	90	80	25	30	Rad.	Normal	12	2	6
13	M	45	0	50	5	90	85	20	30	IL	Her.	8	8	8
14	M	32	1	55	0	75	75	20	35	IL	Her.	10	6	4
15	M	17	0	40	0	90	80	25	30	IL	Her.	8	2	10
16	M	47	2	55	5	75	65	10	20	Rad.	Her. + K-WIRE	16	7	12
17	M	49	1	50	0	65	70	15	25	IL	Her.	12	10	16
18	M	38	0	45	5	80	80	20	30	IL	Her.	8	4	12
19	M	37	1	50	5	75	60	20	25	IL	Her.	10	11	4
20	M	31	0	45	0	90	75	20	30	Rad.	Mini	12	8	10
21	F	21	2	50	5	80	70	20	25	IL	Her.	10	6	12
22	M	36	2	50	0	80	70	25	25	Rad.	Her.	12	3	8
23	F	28	1	45	0	90	85	20	30	Rad.	ACCU	8	2	6

Abreviaturas: ACCU, Accutrak; consol., tempo até a consolidação; ext., extensão; flex., flexão; Her., Herbert; IL, crista ilíaca; mini, parafuso de minifragmentos; rad., rádio; RD, desvio radial; RLA, ângulo radioulnar; SLA, ângulo escafolunar; UD, desvio ulnar.

**Fig. 8:** Paciente com oito semanas de pós-operatório, visão da face dorsal e volar do punho com as incisões dos portais da artroscopia e da fixação percutânea do escafoide (A e B). Recuperação completa da mobilidade, tanto da prono-supinação (C e D), quanto da extensão e flexão (E e F).

## Discussão

Outros estudos já mostraram bons resultados utilizando métodos minimamente invasivos no tratamento das fraturas e PAE. Eles estão associados à menor morbidade, apresentando rápida recuperação e permitindo o tratamento de lesões associadas durante o mesmo procedimento. 6 9 14 17 23

Quando não tratada, a PAE gera instabilidade que progride para um processo degenerativo precoce, com colapso do carpo, padrão conhecido com punho SNAC. 1 5 Assim, o objetivo do tratamento cirúrgico da PAE é obter a consolidação óssea associada à restauração da anatomia óssea e do alinhamento do carpo. Para isso tem sido descritas técnicas com diferentes conceitos, umas que priorizam a estabilidade óssea mecânica, inclusive com utilização de placas e parafusos, e outras que tem como objetivo a manutenção e o estímulo à biologia vascular, estimulando a consolidação precoce. 7 14 16 18 24

A artroscopia foi inicialmente indicada para as PAE estáveis e fibrosas, porém, com a evolução da técnica e a comprovação que não há necessidade de enxerto estrutural para a correção da deformidade em flexão do escafoide, seu uso foi expandido para as PAE instáveis e com colapso em flexão. Nas PAE crônicas e instáveis, as técnicas com uso de enxertos ósseos córtico-esponjosos (vascularizados ou não) são as que oferecem maior garantia de obter-se a consolidação óssea e restaurar o alinhamento e a anatomia do carpo. Porém, por serem técnicas abertas, geram agressão aos tecidos locais e às estruturas neuro-vasculares, podendo também lesar os ligamentos estabilizadores extrínsecos do carpo, além do risco de rigidez, infecção, protrusão do enxerto e demora em obter consolidação completa, tempo que varia na literatura entre 12 a 19 semanas. 2 3 6

Tentando diminuir o trauma aos tecidos locais e melhorar o potencial de consolidação, alguns artigos descrevem bons resultados com o uso de enxerto ósseo esponjoso em PAE instáveis. Park et al. 22 trataram 61 pacientes com PAE, sendo 52% estáveis e 48% instáveis, com uso de enxerto ósseo esponjoso e fixação com fios de Kirschner. Eles obtiveram consolidação em 88,2% das lesões estáveis e 83,9% das instáveis, sem diferenças estatísticas significativas entre os grupos.

Não existe regra quanto do tipo de enxerto ósseo a ser usado. Preferimos o uso enxerto do ilíaco em pacientes com grave deformidade e reabsorção óssea. Já em PAE estáveis e com pouca deformidade utilizamos o enxerto ósseo esponjoso retirado da metáfise distal do rádio. O tempo curto em obter a consolidação, média de 7,5 semanas, é inferior aos apresentados em séries usando a técnica aberta, tanto em enxertos vascularizados como não vascularizados, permitindo pouco tempo de imobilização e retorno precoce às atividades.

Algumas séries mostram resultados semelhantes ao nosso com o uso de artroscopia e fixação percutânea com parafuso, sem usar enxerto ósseo. 9 13 14 23 25 Slade et al. 13 publicaram uma série de 15 pacientes com PAE estáveis obtendo consolidação em todos, em um tempo médio de 14 semanas. Já Chu e Shih 26 trataram 15 pacientes PAE estáveis com artroscopia associada a fixação percutânea de parafuso combinada e uso de matriz óssea desmineralizada. Mostraram 93% de consolidação em 15 semanas de média. Na série de Wong e Ho com 68 pacientes com PAE tratados com a técnica artroscópica e parafusos houve 91,2% de consolidação, em um período médio de 12 semanas, sem apresentar nenhuma complicação decorrente do método. 10 Kim et al. 17 avaliaram 36 pacientes com artroscopia e enxerto ósseo esponjoso para PAE instáveis obtendo consolidação em 86% dos pacientes em uma média de 11 semanas.

Com a técnica artroscópica com uso de enxerto ósseo esponjoso é mais difícil restaurar a anatomia do escafoide e o alinhamento normal do carpo. Porém, outras técnicas também mostram dificuldade semelhante. Jiranek et al. 27 em uma série de pacientes tratados por PAE com enxerto ósseo cortico-esponjoso trapezoidal obtiveram uma deformidade de 45° ou mais em mais no ângulo intra-escafoide em mais da metade dos casos e não encontrou relação do alinhamento com o resultado clínico final. Também da série Kim et al., 17 os parâmetros radiológicos do escafoide e do alinhamento do carpo não se correlacionaram função clínica final do paciente. Assim como esses autores, pensamos que os efeitos positivos da técnica artroscópica e fixação percutânea superam uma possível dificuldade de correção completa da deformidade, sendo que isso não altera a função final do punho. 28

A artroscopia de punho é uma alternativa válida em o tratamento de lesões do escafoide do carpo. Apresenta bons resultados clínicos e de consolidação, mesmo em lesões com deformidade e instabilidade carpal, com um mais curto de recuperação. Provavelmente é limitado para restaurar alinhamento normal do carpo, mas é benéfico e melhora tempo de recuperação para essas lesões.

## Conclusão

Os resultados apresentados demonstram baixa morbidade e recuperação rápida com esta técnica. No entanto, para confirmar essas afirmações e resultados, necessitamos de séries maiores, com acompanhamento mais longo e com pacientes randomizados em grupos, comparando diferentes técnicas.
